# A narrative review on the interplay between interstitial lung disease and obstructive sleep apnea: Mechanisms, clinical implications, and management strategies

**DOI:** 10.1016/j.sleepx.2026.100191

**Published:** 2026-06-24

**Authors:** Nesrin Ocal, Baran Balcan, Yüksel Peker

**Affiliations:** a Health Sciences University, Gulhane Faculty of Medicine, Department of Pulmonology, Ankara, Türkiye; b Koc University, Faculty of Medicine, Department of Pulmonology, Istanbul, Turkiye; c Koc University Research Center for Translational Medicine, Istanbul, Türkiye; d Division of Pulmonary, Allergy, and Critical Care Medicine, University of Pittsburgh School of Medicine, Pittsburgh, USA; e Department of Molecular and Clinical Medicine, Institute of Medicine, Sahlgrenska Academy, University of Gothenburg, Gothenburg, Sweden; f Department of Clinical Sciences, Respiratory Medicine and Allergology, Faculty of Medicine, Lund University, Lund, Sweden

## Abstract

Interstitial lung diseases (ILDs) comprise a heterogeneous group of diffuse parenchymal lung disorders associated with progressive fibrosis, impaired gas exchange, and substantial morbidity. In recent years, the coexistence of obstructive sleep apnea (OSA) in ILD has emerged as an underrecognized yet clinically meaningful overlap. Evidence indicates that OSA is highly prevalent in ILD—even among non-obese patients—and is driven by restrictive mechanics, diminished lung volumes, impaired diaphragmatic function, and severe nocturnal hypoxemia. These physiological factors create a unique OSA phenotype that differs substantially from classic presentations observed in the general population.

A growing body of data suggests that the interplay between ILD and OSA is not incidental but intertwined. Intermittent hypoxia, recurrent arousals, and autonomic activation have been proposed as mechanisms that may contribute to adverse clinical phenotypes in ILD; however, available evidence remains largely observational, and the independent causal contribution of OSA beyond ILD severity and nocturnal hypoxemia remains uncertain., REM-related oxygen instability, and marked sleep fragmentation—findings that may remain undetected by standard screening tools or home sleep apnea testing.

Recognition of OSA in ILD is clinically important because PAP therapy, supplemented by oxygen when needed, may improve sleep quality, fatigue, daytime symptoms, and nocturnal oxygenation; whether this translates into slower ILD progression or improved survival remains unproven. Management requires individualized strategies that account for restrictive physiology, ventilatory instability, and the high burden of hypoxemia.

This review synthesizes current evidence on the epidemiology, mechanisms, clinical implications, and management of OSA in ILD, highlighting the need for early screening, precise diagnostic evaluation, and tailored treatment approaches. Key knowledge gaps and priorities for future research are also outlined to guide the development of integrated, multidisciplinary care strategies for this complex patient population.

## Highlights

• OSA is common but frequently underrecognized in patients with ILD.
• ILD-related restrictive physiology promotes a distinct sleep-disordered breathing phenotype.
• Severe nocturnal desaturation may occur despite relatively modest OSA severity.
• PSG remains the preferred diagnostic approach in ILD patients with suspected OSA.
• Early recognition and individualized treatment of OSA may improve patient-centered outcomes.

## Graphical abstract

**Figure:**
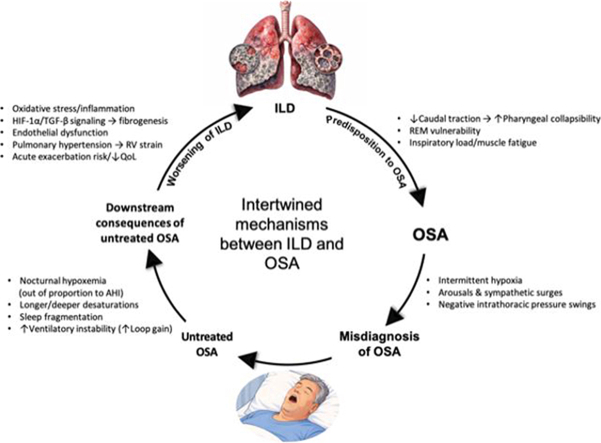

## 1 Introduction

Interstitial lung diseases (ILDs) represent a heterogeneous group of diffuse parenchymal lung disorders characterized by varying degrees of inflammation, fibroproliferation, and architectural distortion. Among these, idiopathic pulmonary fibrosis (IPF), connective tissue disease–associated ILDs (CTD-ILDs), hypersensitivity pneumonitis (HP), and sarcoidosis constitute the most clinically relevant subgroups. ILDs collectively impose a substantial burden owing to progressive respiratory impairment, chronic hypoxemia, reduced functional capacity, and impaired health-related quality of life. While their pulmonary manifestations are well recognized, the systemic consequences of ILDs particularly their effects on sleep physiology have gained prominence only recently.

Obstructive sleep apnea (OSA), defined by recurrent episodes of upper-airway collapse during sleep, affects an estimated 10–30% of adults in the general population, with prevalence increasing with aging and obesity [1,2]. Beyond classic risk factors, emerging evidence suggests that OSA is highly prevalent among patients with ILD, even in those with normal or near-normal body mass index (BMI) [3]. Unlike traditional OSA populations, ILD patients often exhibit unique phenotypes mediated by restrictive physiology, diaphragmatic weakness, impaired lung mechanics, and profound nocturnal hypoxemia [3]. These patterns suggest that the coexistence of ILD and OSA is not coincidental but rather reflects shared physiological and possibly pathogenic pathways.

The intersection of ILD and OSA represents a clinically significant overlap with implications for disease progression, exacerbations, pulmonary hypertension, cardiovascular comorbidity, and mortality [3,4]. Nocturnal hypoxemia a hallmark feature of both OSA and ILD—may play a key role in accelerating fibrogenesis through hypoxia-inducible pathways, oxidative stress, vascular remodeling, and autonomic dysregulation [5,6]. Furthermore, OSA-related sleep fragmentation exacerbates fatigue and reduces exercise capacity, amplifying symptom burden in ILD patients.

**Fig. 1:**
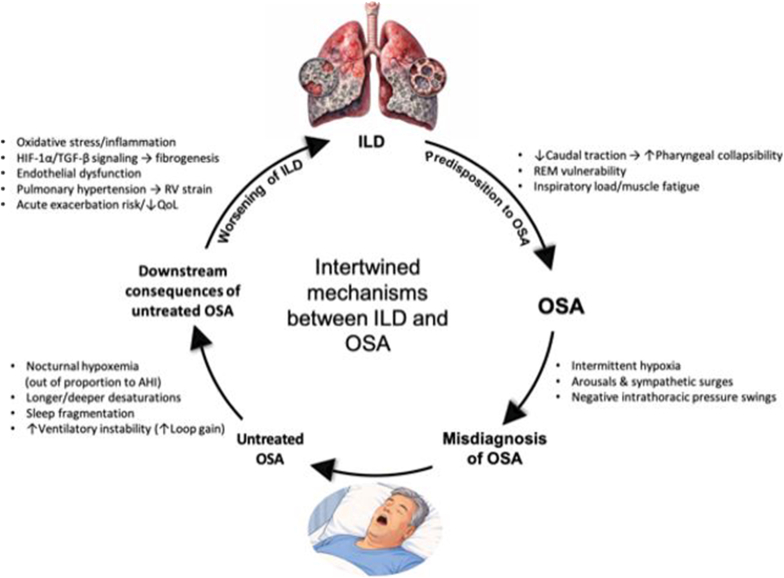
Intertwined pathophysiological mechanisms linking interstitial lung disease and obstructive sleep apnea. Definition of abbreviations: ILD = interstitial lung disease; OSA = obstructive sleep apnea; AHI = apnea–hypopnea index; HIF-1α = hypoxia-inducible factor-1 alpha; RV = right ventricle; REM = rapid eye movement.

Despite increasing recognition of this overlap, current guidelines offer limited direction on screening, diagnostic evaluation, or treatment strategies. Variability in disease expression, limited inclusion of ILD patients in large OSA studies, and the lack of mechanistic trials contribute to this knowledge gap. Given the growing evidence of clinical relevance, a comprehensive synthesis is needed to clarify epidemiologic patterns, elucidate mechanistic underpinnings, examine outcome data, and propose practical management strategies (Fig. 1).

This review provides an up-to-date, in-depth exploration of the interplay between ILD and OSA, drawing on epidemiologic data, translational research, and clinical studies. We discuss shared and distinct pathophysiologic mechanisms, polysomnographic features, prognostic implications, and therapeutic approaches, culminating in a proposed clinical algorithm and future research priorities.

## 2 Epidemiology of OSA in ILD

Over the past decade, multiple studies have documented a disproportionately high prevalence of OSA across ILD subtypes [6,7]. Early reports suggested that up to 50–70% of IPF patients exhibit clinically significant OSA, with more recent data indicating even higher rates when nocturnal hypoxemia is included as part of the phenotype [4]. Importantly, OSA in ILD is frequently underrecognized because its clinical presentation differs from that in classic OSA populations (Table 1).

**Table 1:** Clinical, epidemiologic, polysomnographic, prognostic, and therapeutic evidence on OSA in ILD.

Outcome	Year	Main findings	Author et al. [Ref #]
Prevalence of OSA in ILD (meta-analysis)	2021	High OSA prevalence across ILDs	Cheng et al. [3]
OSA in fibrotic ILD (non-IPF)	2024	OSA common in fibrotic ILD	Cardoso et al. [4]
OSA in chronic respiratory disease	2022	OSA frequent in restrictive disease	Locke et al. [6]
OSA prevalence in IPF (systematic review)	2024	Very high OSA prevalence	Wei et al. [7]
OSA risk in systemic sclerosis	2021	Multisystem factors increase OSA	Saketkoo et al. [8]
OSA in Sjögren's syndrome	2022	High OSA frequency observed	Karabul et al. [9]
Sleep disorders in chronic HP	2023	Poor sleep, frequent SDB	Martins et al. [10]
Sleep disruption in hypersensitivity pneumonitis	2024	Sleep impairment common	Mariano & Genta [11]
Sleep-disordered breathing in IPAF	2024	OSA common in autoimmune ILD	Joerns & Sparks [12]
Sleep-related hypoventilation in PPFE	2022	Nocturnal hypoventilation frequent	Yabuuchi et al. [13]
Lymphoid interstitial pneumonia	2025	Severe gas-exchange impairment	Lau et al. [14]
Overnight desaturation in ILD	2024	Linked to mortality and vasculopathy	Margaritopoulos et al. [15]
Sleep in restrictive lung disease	1986	Rapid shallow breathing during sleep	George & Kryger [16]
Nocturnal hypoxemia in ILD	2021	Hypoxemia precedes daytime oxygen	Khor et al. [17]
OSA in idiopathic pulmonary fibrosis	2009	OSA common, non-obese	Lancaster et al. [18]
Sleep-disordered breathing in ILD	2014	Underrecognized clinical problem	Troy & Corte [19]
REM-related OSA	2024	REM sleep increases vulnerability	Bonsignore et al. [20]
OSA in fibrotic ILD and COPD	2019	High OSA prevalence	Zhang et al. [21]
Predictors of sleep-disordered breathing	2024	Hypoxemia predicts OSA	Laz et al. [22]
Nocturnal desaturation index	2012	Predicts mortality	Corte et al. [23]
Nocturnal desaturation patterns in IPF	2021	Severe oxygen dips during sleep	Yasuda et al. [24]
Sleep architecture in ILD with PH	2022	Reduced REM, high arousals	Simonson et al. [25]
Sleep disorders in IPF	2016	Marked sleep fragmentation	Milioli et al. [26]
Respiratory patterns during sleep	2013	Persistent tachypnea observed	Mermigkis et al. [27]
Sleep in interstitial lung disease	2009	OSA and hypoxemia common	Agarwal et al. [28]
OSA and prognosis in ILD	2023	OSA predicts worse outcomes	Valecchi et al. [29]
OSA and pulmonary hypertension	2024	Bidirectional pathophysiology	Balcan et al. [30]
Autonomic dysfunction in OSA	2014	Increased sympathetic activity	Abboud & Kumar [31]
Comorbidity clusters in fibrotic ILD	2020	OSA linked to worse phenotype	Wong et al. [32]
Pulmonary hypertension in ILD	2022	Major prognostic factor	Dhont et al. [33]
Pulmonary hypertension prevalence in ILD	2024	Common and prognostically adverse	Ang et al. [34]
OSA-related pulmonary hypertension	2021	Hypoxia-driven vascular changes	Shah et al. [35]
Sleep and pulmonary hypertension	2020	Sleep worsens hemodynamics	Louise et al. [36]
Noninvasive PH prediction in IPF	2019	Sleep indices included	Sonti et al. [37]
Nocturnal hypoxemia and mortality	2023	SpO_2_<90% predicts death	Myall et al. [38]
CPAP effects on pulmonary artery pressure	2014	CPAP reduces PAP	Sun et al. [39]
CPAP effects on PH (RCT)	2006	PAP improves hemodynamics	Arias et al. [40]
CPAP plus oxygen in IPF	2024	Improved nocturnal oxygenation	Bordas-Martinez et al. [41]
PAP treatment in IPF with OSA	2021	Better survival with adherence	Papadogiannis et al. [42]
CPAP adherence and ILD survival	2020	Improved progression-free survival	Adegunsoye et al. [43]
Sleep as therapeutic target in IPF	2017	Treating sleep may improve outcomes	Mermigkis et al. [44]
PAP therapy guidelines	2019	Improves sleep and oxygenation	Patil et al. [45]
CPAP plus oxygen feasibility	2024	Safe and effective	Bordas-Martinez et al. [46]
CPAP difficulties in IPF	2012	Adherence challenges common	Mermigkis et al. [47]
Supplemental oxygen in ILD	2023	Improves nocturnal hypoxemia	Clark et al. [48]
Pulmonary rehabilitation in ILD	2014	Improves functional capacity	Dowman et al. [49]

Definition of abbreviations: AHI = apnea–hypopnea index; BMI = body mass index; COPD = chronic obstructive pulmonary disease; CPAP = continuous positive airway pressure; CTD = connective tissue disease; HP = hypersensitivity pneumonitis; HSAT = home sleep apnea testing; ILD = interstitial lung disease; IPAF = interstitial pneumonia with autoimmune features; IPF = idiopathic pulmonary fibrosis; NIV = noninvasive ventilation; OSA = obstructive sleep apnea; PAP = positive airway pressure; PAP = pulmonary artery pressure; PH = pulmonary hypertension; PPFE = pleuro-parenchymal fibroelastosis; PSG = polysomnography; RCT = randomized controlled trial; REM = rapid eye movement; SDB = sleep-disordered breathing; SpO_2_ = peripheral capillary oxygen saturation.

### 2.1 Idiopathic pulmonary fibrosis (IPF)

IPF has the strongest epidemiologic link with OSA among ILDs [7]. Prevalence estimates range from 60% to 90%, depending on diagnostic thresholds and cohort characteristics. Notably, obesity—one of the strongest traditional risk factors for OSA—is often absent in IPF cohorts. Instead, reductions in lung volume and compliance appear to predispose these patients to upper-airway collapsibility independent of BMI [50]. Progressive fibrosis results in diminished caudal traction on the upper airway, enhancing pharyngeal collapsibility and contributing to obstructive events [50]. Moreover, IPF patients frequently manifest pronounced nocturnal desaturation out of proportion to the AHI, highlighting the central role of gas-exchange impairment [51].

### 2.2 Connective tissue Disease–Associated ILD

CTD-ILDs, including rheumatoid arthritis–associated ILD, systemic sclerosis (SSc-ILD), Sjögren's-related ILD, and inflammatory myositis–associated ILD, also demonstrate elevated rates of OSA [8,9]. Several factors contribute: restrictive physiology, diaphragmatic dysfunction, small-airway involvement, and systemic inflammation. In SSc-ILD, microaspiration, esophageal dysmotility, and upper-airway stiffness may further increase vulnerability to sleep-disordered breathing [8].

### 2.3 Hypersensitivity pneumonitis (HP) and other ILDs

Hypersensitivity pneumonitis (HP), particularly chronic fibrotic HP, appears to fall within the intermediate-to-high range of OSA prevalence reported across interstitial lung diseases. In mixed ILD cohorts that include HP and other non-IPF forms, approximately half of patients are found to have OSA, supporting the concept that OSA is common beyond IPF alone [3]. A recent study specifically examining sleep in chronic HP has shown poorer sleep quality, a high burden of sleep-related symptoms, and a signal for sleep-disordered breathing, prompting calls for systematic screening in this population [10]. Chronic antigen exposure and the development of fibrotic HP frequently result in a combination of airflow limitation and restrictive mechanics, patterns that mirror those described as predisposing to OSA in other fibrotic ILDs [11].

Unclassifiable ILD and related categories, such as interstitial pneumonia with autoimmune features (IPAF), constitute a substantial minority of ILD cases and often share similar comorbidity profiles, including a notable burden of sleep-disordered breathing and OSA [12]. Organizing pneumonia, pleuroparenchymal fibroelastosis (PPFE), and lymphoid interstitial pneumonia (LIP) remain much less well studied from a sleep perspective; however, available data indicate that PPFE is associated with marked restrictive physiology, early sleep-related hypoventilation, and hypercapnia, features that are likely to interact with sleep-disordered breathing [13]. LIP is similarly characterized as a rare interstitial pneumonia with diffuse parenchymal involvement and impaired gas exchange, suggesting a physiologic predisposition to nocturnal hypoxemia even though formal OSA prevalence studies are lacking [14].

## 3 Shared and distinct pathophysiology

Understanding the mechanistic interplay between ILD and OSA requires integrating insights from pulmonary mechanics, neural control of ventilation, inflammatory pathways, and cardiovascular physiology. The relationship is bidirectional: ILD may predispose individuals to OSA, while OSA-related hypoxia and mechanical stress may accelerate fibrogenesis or worsen ILD outcomes (Table 2).

**Table 2:** Mechanistic and pathophysiologic evidence linking ILD and OSA.

Outcome	Year	Main findings	Author et al. [Ref #]
Upper airway–lung interaction	2024	Reduced lung volume increases collapse	Sunwoo & Malhotra [50]
End-expiratory lung volume during sleep	2017	Reduced lung volume worsens OSA	Koo et al. [51]
Upper airway pathophysiology	2008	Lung volume stabilizes airway	Eckert & Malhotra [52]
Sleep-disordered breathing in neuromuscular disease	2017	Muscle weakness worsens SDB	Aboussouan & Cabodevila [53]
Sleep disorders in neuromuscular disease	2023	Hypoventilation common	Boentert [54]
Ventilatory instability (loop gain)	2024	Hypoxia increases loop gain	Antonaglia et al. [55]
Intermittent hypoxia and inflammation	2005	Activates fibrogenic pathways	Ryan et al. [56]
Inflammatory biomarkers in OSA	2016	Elevated oxidative stress markers	Dogan et al. [57]
Oxidative stress in OSA	2003	Hypoxia–reoxygenation injury	Lavie [58]
Endothelial dysfunction in OSA	2008	Reduced vascular repair capacity	Jelic et al. [59]
Endothelial effects of OSA	2021	Promotes vascular remodeling	Mochol et al. [60]
OSA diagnostic guidelines	2017	PSG preferred over HSAT	Kapur et al. [61]
Noninvasive ventilation guidelines	2017	NIV role in respiratory failure	Rochwerg et al. [62]
Antifibrotic therapy in IPF	2014	Slows disease progression	Richeldi et al. [63]
CTD-ILD treatment overview	2012	Immunosuppression impacts	Fischer & du Bois [64]

CTD = connective tissue disease; HSAT = home sleep apnea testing; ILD = interstitial lung disease; IPF = idiopathic pulmonary fibrosis; NIV = noninvasive ventilation; OSA = obstructive sleep apnea; PSG = polysomnography; REM = rapid eye movement; SDB = sleep-disordered breathing.

### 3.1 Mechanical factors

#### 3.1.1 Loss of lung volume and caudal traction

ILD is characterized by parenchymal fibrosis and reduced lung compliance. This leads to decreased functional residual capacity (FRC) and diminished caudal traction on the upper airway [15]. Under normal circumstances, lung inflation increases longitudinal tension that stabilizes the pharyngeal lumen [52]. Loss of this stabilizing force in ILD promotes pharyngeal collapse during sleep—a mechanism supported by physiologic experiments demonstrating increased upper-airway collapsibility in restrictive lung diseases. Fibrosis, chest wall rigidity, and altered thoracoabdominal mechanics contribute to reduced tidal volumes and higher respiratory rates during sleep [16]. These factors increase the propensity for upper airway collapse, particularly during REM sleep when muscle atonia diminishes compensatory airway-dilating tone [53]. Diaphragmatic weakness, whether due to systemic autoimmune disease, steroid-induced myopathy, or chronic hyperinflation, disrupts sleep stability [53]. Inspiratory muscle fatigue promotes periodic breathing and increases negative intrathoracic pressure swings, thereby worsening upper-airway collapsibility [54].

### 3.2 Gas-exchange abnormalities and ventilatory control

Chronic gas-exchange impairment is one of the defining features of ILD and significantly effects sleep physiology. Due to loss of alveolar–capillary surface area, thickened interstitium, and ventilation–perfusion mismatch, ILD patients often exhibit isolated nocturnal hypoxemia even before daytime oxygen requirements emerge [17]. During sleep—especially REM sleep—hypoventilation, reduced respiratory drive, decreased accessory muscle recruitment, and worsened V/Q matching amplify oxygen desaturation.

OSA further magnifies this vulnerability. The cyclical pattern of obstructive apneas induces intermittent hypoxia and hypercapnia, triggering oscillations in ventilatory drive. ILD patients have heightened chemosensitivity and elevated loop gain due to chronic hypoxia, increasing the risk of unstable breathing patterns [55]. This predisposes them to more severe desaturation per apnea event and more frequent arousals, reinforcing a vicious cycle of sleep fragmentation, sympathetic activation, and inflammation.

### 3.3 Inflammation, oxidative stress, and fibrogenesis

Both ILD and OSA are characterized by heightened systemic inflammation. Repeated cycles of intermittent hypoxia generate oxidative stress, reactive oxygen species (ROS), and activation of key fibrogenic pathways including transforming growth factor-beta (TGF-β), hypoxia-inducible factor-1α (HIF-1α), and nuclear factor kappa B (NF-κB) [56,57]. Intermittent hypoxia increases fibroblast proliferation, epithelial–mesenchymal transition, and extracellular matrix deposition [56].

This raises the hypothesis that untreated OSA may accelerate ILD progression, particularly in fibrotic phenotypes such as IPF [18]. Although mechanistic trials are lacking, observational data demonstrate that patients with coexisting OSA have more rapid declines in forced vital capacity (FVC) and diffusing capacity (DLCO), as well as more frequent acute exacerbations [29]. Chronic intermittent hypoxia may also exacerbate microvascular injury, contributing to pulmonary hypertension—a known adverse prognostic marker in ILD.

### 3.4 Cardiovascular crosstalk and autonomic instability

Pulmonary hypertension (PH) is prevalent in advanced ILD and is strongly associated with poor outcomes. OSA independently contributes to PH through hypoxic pulmonary vasoconstriction, endothelial dysfunction, increased sympathetic tone, and impaired nitric oxide signaling [30]. When OSA coexists with ILD, these mechanisms act synergistically, placing substantial strain on the right ventricle.

Furthermore, autonomic dysregulation—characterized by increased sympathetic outflow and reduced parasympathetic tone—may worsen nocturnal dyspnea and sleep fragmentation [31]. The heightened adrenergic state persists during the daytime, contributing to hypertension, arrhythmias, and cardiovascular morbidity. This overlap underscores the importance of early identification and management of sleep-disordered breathing in ILD populations.

### 3.5 Nighttime physiology: sleep architecture and arousal burden

Sleep disruption is nearly universal in ILD. Persistent cough, dyspnea, steroid effects, anxiety, and gastroesophageal reflux lead to frequent nighttime awakenings. Polysomnography studies reveal reduced slow-wave sleep, reduced REM sleep, increased stage N1 sleep, and elevated arousal indices [19].

OSA superimposed on ILD further distorts sleep architecture. REM sleep—normally a period of physiological vulnerability—is characterized by atonia of airway dilator muscles and increased upper airway resistance [20]. In ILD patients, REM-related desaturation is often profound, with oxygen saturations dropping below 80% even with relatively low AHI [19]. This phenomenon is clinically significant because REM-predominant OSA has been linked to greater sympathetic surges, arrhythmogenic risk, endothelial dysfunction, and impaired cardiometabolic health.

## 4 Clinical manifestations and screening

### 4.1 Atypical symptomatology

The classical symptoms of obstructive sleep apnea, such as loud snoring, witnessed apneas, and excessive daytime sleepiness, tend to be less prominent in patients with interstitial lung disease. Instead of presenting with the typical picture, these patients more commonly describe a persistent sense of fatigue rather than true sleepiness [18]. They may experience morning headaches and report that their sleep does not feel refreshing despite an adequate duration. Many also struggle with exertional dyspnea, which can mask or overshadow sleep-related breathlessness, making nocturnal symptoms more difficult to recognize. Cognitive slowing and mood disturbances are frequently noted as well, further contributing to their overall symptom burden.

Daytime sleepiness assessed using the Epworth Sleepiness Scale is often within the normal range in individuals with ILD, which limits the utility of this tool for screening. Moreover, many patients underreport symptoms of sleep apnea because they assume their fatigue and reduced daily activity levels are solely a consequence of their underlying lung disease. This tendency contributes to underdiagnosis and highlights the need for heightened clinical suspicion in this population [18] (Table 1).

### 4.2 Screening tools and their limitations

Common screening tools such as STOP-BANG, the Berlin Questionnaire, and NoSAS show reduced accuracy in patients with interstitial lung disease, especially in those who are not obese. This limitation arises because BMI-based scoring tends to underestimate risk, and factors such as older age and multiple comorbidities can confound the interpretation of results [65]. In addition, ILD patients often exhibit atypical sleepiness patterns, making subjective assessments less reliable. The restrictive nature of the disease also contributes to a higher rate of false-negative findings, further diminishing the sensitivity of these tools. Despite these challenges, a STOP-BANG score of three or higher can still identify a moderate proportion of ILD patients with clinically significant obstructive sleep apnea, although its specificity remains low [21].

### 4.3 Who should Be screened?

Given the high burden and clinical consequences of sleep-disordered breathing in interstitial lung disease, screening should be strongly considered in patients who present with resting or exertional hypoxemia, or who report unexplained fatigue that appears disproportionate to the degree of lung function impairment [19]. Screening is also appropriate in individuals with pulmonary hypertension, in those who demonstrate disproportionate nocturnal desaturation on overnight oximetry, or who show REM-related oxygen drops on home sleep testing. Clinical concern raised by bed partners, as well as the presence of CTD-ILD or with systemic manifestations, should similarly prompt evaluation. In this population, the threshold for proceeding to full polysomnography should be low [22].

### 4.4 Diagnostic evaluation

Polysomnography remains the gold standard for evaluating sleep-disordered breathing in patients with interstitial lung disease because home sleep apnea testing frequently underestimates the apnea–hypopnea index in individuals with significant hypoxemia or other comorbid lung conditions [61]. In addition, home-based tests provide imprecise detection of REM sleep and often fail to identify hypoventilation, which is particularly relevant in restrictive disorders. They also do not reliably capture clinically critical parameters such as the oxygen saturation nadir and the time spent below 90 percent saturation, both of which carry prognostic significance in ILD [23]. When PSG findings are borderline or inconclusive, further assessment using adjunctive tools—such as transcutaneous carbon dioxide monitoring, esophageal pressure measurements, or simultaneous cardiopulmonary monitoring—may be necessary to fully characterize sleep-related physiologic disturbances.

## 5 Polysomnographic characteristics in ILD with OSA

The PSG phenotype in ILD patients with OSA differs significantly from that seen in classic OSA populations (Table 1).

### 5.1 Oxygen desaturation disproportionate to AHI

The defining feature of sleep-related breathing disturbances in ILD is the presence of profound nocturnal desaturation. Even modest obstructive sleep apnea—with AHI values in the mild range—may lead to marked oxygen drops because of the underlying diffusion impairment and reduced pulmonary reserve characteristic of ILD [19]. In many patients, oxygen saturation can fall to very low levels during sleep, and polysomnographic studies frequently demonstrate substantial periods of time with SpO_2_<90%, reflecting the combined effects of ventilation–perfusion mismatch and restrictive mechanics [24].

In addition to deep desaturation events, several other oxygenation patterns are typical in ILD, including REM-related oxygen declines that may occur even in the absence of clear obstructive events, and a flattened overnight saturation profile driven by diffusion limitation and the loss of pulmonary capillary surface area [19]. These physiologic abnormalities underscore the vulnerability of ILD patients to even mild forms of sleep-disordered breathing and highlight the importance of comprehensive overnight monitoring in this population.

### 5.2 Sleep architecture alterations

Sleep architecture in patients with interstitial lung disease and coexisting obstructive sleep apnea is frequently and significantly disrupted. These individuals commonly exhibit reduced REM sleep, along with an increased proportion of stage N1 sleep, reflecting a lighter and more fragmented sleep pattern [25]. Slow-wave sleep is typically diminished, and the arousal index is often markedly elevated, frequently exceeding twenty to thirty arousals per hour. As a result of these disturbances, overall sleep efficiency tends to fall below seventy percent in many cases. Patients with usual interstitial pneumonia and other fibrotic patterns generally demonstrate the most pronounced alterations in sleep architecture [25,26].

### 5.3 Respiratory patterns

Respiratory patterns during sleep are also distinctly abnormal in this population. Polysomnographic and physiologic studies in ILD show frequent respiratory-related arousals and a predominance of rapid, shallow breathing that persists during non-REM sleep, rather than slowing as in healthy individuals [27]. This altered pattern reflects the increased elastic load and impaired gas exchange of restrictive lung disease, and contributes to sleep fragmentation and non-apneic desaturation events [19]. In ILD cohorts, apneas and hypopneas are often numerically modest overall, but central and mixed events have been reported, particularly in patients with more severe nocturnal hypoxemia and advanced parenchymal disease [28]. Ventilation–gas exchange studies also demonstrate that tachypnea and elevated ventilatory drive persist into non-REM sleep, indicating that respiratory rate and effort remain inappropriately high at night as a compensatory response to diffusion limitation and reduced lung volumes [27]. Together, these abnormalities underline the substantial physiologic strain imposed by ILD on sleeping respiration and help explain the high burden of sleep disruption in this group.

### 5.4 Coexisting sleep disorders

Periodic limb movement disorder is reported in 20–30% of ILD patients and may further fragment sleep. Insomnia symptoms are common and often related to cough, dyspnea, and anxiety [19]. These disorders frequently coexist with OSA and complicate the interpretation of daytime symptoms.

## 6 Impact of OSA on ILD outcomes

The coexistence of OSA in patients with ILD is more than a simple overlap; it represents a clinically meaningful interaction with measurable effects on disease trajectory, symptom burden, and survival. Observational cohorts suggest that OSA and nocturnal hypoxemia are associated with worse clinical status and outcomes in ILD; however, these associations may partly reflect more advanced parenchymal disease, impaired gas exchange, pulmonary vascular involvement, and other residual confounders (Table 1).

### 6.1 Disease progression and lung function decline

Growing evidence suggests that obstructive sleep apnea may accelerate disease progression in interstitial lung disease, especially in fibrotic forms. Intermittent hypoxia can stimulate fibroblast activation through increased TGF-β signaling and epithelial–mesenchymal transition, while repeated hypoxia–reoxygenation cycles promote oxidative stress and mitochondrial injury [56,58]. OSA-related endothelial dysfunction may further worsen the microvascular impairment already present in ILD [59]. Clinically, patients with moderate to severe OSA often show faster declines in FVC and DLCO and more frequent radiologic progression. Although causality has not been definitively established, these findings support the concept that untreated OSA amplifies ongoing fibrogenesis [32].

### 6.2 Pulmonary hypertension and right ventricular dysfunction

Pulmonary hypertension (PH) is a major determinant of prognosis in interstitial lung disease (ILD), and its presence is associated with worse exercise capacity, greater oxygen requirements, and reduced survival compared with ILD without PH [33,34]. When OSA coexists, several pathophysiologic mechanisms can further aggravate pulmonary vascular load: recurrent nocturnal hypoxemia drives hypoxic pulmonary vasoconstriction, while oxidative stress and systemic inflammation contribute to endothelial dysfunction, reduced nitric oxide bioavailability, and pulmonary vascular remodeling [35,60]. Large negative intrathoracic pressure swings during obstructive events also increase right ventricular (RV) afterload and wall stress, and OSA has been linked to subclinical RV dysfunction in echocardiographic studies [36]. In ILD cohorts, OSA is common and frequently overlaps with PH, and non-invasive prediction models for PH in IPF already incorporate parameters related to sleep-disordered breathing and gas-exchange impairment [37]. Although most interventional data come from non-ILD populations, meta-analyses and controlled studies indicate that CPAP therapy can reduce pulmonary artery pressures in patients with OSA-related PH [39,40], and emerging reports in IPF suggest that effective treatment of coexisting OSA may help stabilize hemodynamics and RV function, supporting a low threshold for diagnosing and treating OSA in ILD patients with suspected or established PH [66].

### 6.3 Mortality

Nocturnal hypoxemia is one of the strongest prognostic markers in interstitial lung disease. Across IPF and Non-IPF ILD cohorts, indices such as the percentage of sleep time with SpO_2_ < 90% and the nocturnal desaturation index independently predict mortality, even after adjustment for age, lung function, and daytime oxygenation [23,38,41]. OSA often deepens and prolongs these desaturation events, and in IPF the combination of OSA and prolonged sleep time with SpO_2_<90% has been shown to be a strong predictor of poor outcome (death or transplantation) [38]. In a broader fibrotic-ILD cohort without daytime hypoxemia, prolonged nocturnal hypoxemia (≥10% of sleep with SpO_2_<90%)—but not OSA defined solely by AHI—was associated with a marked increase in 1-year all-cause mortality, underlining that the depth and duration of desaturation are more prognostically relevant than event frequency [38]. Similar findings from several ILD series indicate that both exertional desaturation and higher AHI have independent negative prognostic significance, supporting a synergistic contribution of daytime and nocturnal gas-exchange impairment to disease progression and death [29].

Data on whether treating OSA improves survival are still limited but suggest potential benefits in selected subgroups. In a prospective IPF cohort, patients with concomitant OSA who achieved ≥4–6 h per night of positive airway pressure (PAP) use had significantly better survival than those with poorer adherence [42]. In a larger multicenter ILD–OSA cohort, overall CPAP adherence did not change mortality, but among patients requiring supplemental oxygen, good CPAP adherence was associated with improved progression-free survival [43]. Taken together, these data support routine assessment of nocturnal oxygenation and OSA in ILD and suggest that aggressive management of sleep-disordered breathing—especially when nocturnal hypoxemia is prominent—should be considered an integral part of strategies to reduce mortality risk in this population.

## 7 Management strategies

Positive airway pressure (PAP) remains the primary therapy for OSA in ILD, improving sleep quality and nocturnal oxygenation, and is associated with better progression-free survival in advanced cases requiring oxygen; however, adherence is often difficult due to dyspnea, cough, anxiety, and rapid desaturation, making careful mask selection, humidification, and close follow-up essential. Bilevel modes or volume-assured pressure support may be needed when hypoventilation, respiratory muscle weakness, or CPAP intolerance is present, and coordinating PAP with supplemental oxygen is critical for patients with severe diffusion impairment. Non-PAP options such as positional therapy, weight optimization, oral appliances, and management of comorbidities offer supportive benefit but have limited effect due to ILD-related restrictive mechanics. Finally, antifibrotic drugs, immunosuppressive regimens, and pulmonary rehabilitation all influence sleep and may impact PAP tolerance, emphasizing the need for integrated sleep-ILD management (Table 1).

### 7.1 Positive airway pressure (PAP) therapy

The management of OSA in ILD requires an individualized and multidisciplinary approach that takes into account restrictive lung mechanics, the severity of hypoxemia, the presence of pulmonary hypertension, and the patient's vulnerability to disturbances in sleep architecture. Contemporary reviews emphasize that standard OSA treatment algorithms often need adaptation in ILD because of unique physiologic challenges, comorbidity patterns, and symptom profiles in this population [5,44].

The PAP therapy remains the primary treatment for OSA in ILD, but applying it in this setting involves specific considerations. The CPAP effectively reduces upper-airway collapse and stabilizes breathing patterns, leading to fewer arousals, improved sleep continuity, and attenuation of nocturnal oxygen desaturation, consistent with data from large OSA populations [45]. In IPF and other fibrotic ILDs, small clinical series have shown that treating coexisting OSA with CPAP can improve sleep-related quality of life, fatigue, and daytime sleepiness, and may favorably influence overall outcomes, although randomized controlled trials are lacking [4,27,42]. In a multicenter ILD cohort with OSA, CPAP adherence was associated with better progression-free survival in the subgroup requiring supplemental oxygen, raising the possibility that selected patients with advanced disease and substantial nocturnal hypoxemia may derive greater clinical benefit from integrated PAP and oxygen-based management, although this remains hypothesis-generating. [43]. A recent pilot study combining CPAP with nocturnal oxygen in IPF further demonstrated good feasibility and improvements in sleep-disordered breathing indices, supporting the practicality of PAP-based strategies in this fragile group [41].

Despite these benefits, adherence is often challenging in ILD. Patients frequently report dyspnea, air hunger, cough, chest wall discomfort, and anxiety or claustrophobia, and clinicians have described substantial therapeutic difficulties when initiating CPAP in IPF [46]. In advanced fibrosis, rapid desaturation during mask removal may add additional stress and limit tolerance. To optimize adherence, experts recommend combining ILD-specific symptom control with standard PAP-adherence strategies—such as heated humidification, careful mask selection (for example, nasal pillows rather than tight full-face masks), gradual desensitization, and close follow-up as outlined in PAP guidelines [45,46]. Coordinating PAP titration with supplemental oxygen and adjusting flow rates to maintain adequate nocturnal SpO_2_ are particularly important in patients with severe diffusion impairment or coexistent pulmonary hypertension [5,47].

In selected cases, bilevel positive airway pressure or advanced modes such as volume-assured pressure support may be indicated—particularly when obstructive events coexist with hypoventilation, when respiratory muscle weakness or chest-wall restriction contributes to chronic hypercapnic respiratory failure (for example, in CTD-ILD with myositis), or when CPAP intolerance persists despite optimization. Although high-quality ILD-specific data are limited, reviews of chronic respiratory failure in ILD and general NIV guidelines suggest that non-invasive ventilation can be used for symptom palliation and gas-exchange support in end-stage restrictive disease, provided that careful titration is used to avoid excessive pressures and potential barotrauma [62,67]. These considerations underline the need to integrate sleep-medicine expertise into ILD care pathways and to individualize PAP modality and settings according to lung mechanics, gas-exchange profile, and patient tolerance.

### 7.2 Supplemental oxygen therapy

Supplemental oxygen therapy plays a key role in the management of nocturnal hypoxemia in ILD patients, including those with coexistent OSA. It is indicated when significant desaturation persists despite adequate OSA treatment, when severe REM-related desaturation occurs independently of obstructive events, when diffusion impairment is pronounced, when pulmonary hypertension is present, or when patients desaturate rapidly during brief mask removal. Although oxygen does not treat upper-airway collapse, it improves nocturnal oxygenation, reduces the cardiovascular burden of hypoxemia, and may blunt sympathetic surges associated with repeated desaturation-reoxygenation cycles. Short-term studies in ILD document that low-flow supplemental oxygen (1–3 L/min) can correct nocturnal hypoxemia and improve heart-rate responses during sleep [17,48]. Combining overnight oxygen with CPAP is often necessary in ILD and may improve sleep quality, stabilize nocturnal saturation, and enhance cardiovascular physiology although long-term survival data are lacking. In ILD-OSA cohorts, good CPAP adherence in patients also on supplemental oxygen has been associated with better progression-free survival [43]. However, the risk of CO_2_ retention should be monitored, and excessively high flow rates or inappropriate mask interface may lead to drying, mucosal irritation or discomfort.

### 7.3 Non-PAP therapies

Non-PAP therapies can also support management, although evidence specific to ILD is limited. Positional therapy may benefit patients with supine-predominant OSA, though its effectiveness diminishes in advanced disease. Weight management remains useful even in non-obese ILD patients, as optimizing body weight can reduce upper-airway resistance and modestly improve mechanics. Oral appliances may help in mild OSA but are generally less effective in ILD, where respiratory mechanics rather than craniofacial anatomy drive airway collapse. Treating comorbidities is essential: gastroesophageal reflux, which is common in IPF, can worsen nighttime symptoms; cough can fragment sleep and reduce CPAP tolerance; and pulmonary hypertension requires early detection and may respond favorably when sleep-disordered breathing is improved.

### 7.4 ILD-specific considerations

ILD-specific therapies also influence sleep. Anti-fibrotic agents such as nintedanib and pirfenidone slow disease progression but may exacerbate gastrointestinal discomfort or disrupt sleep, necessitating monitoring [63]. Immunosuppressive regimens used in CTD-ILD, including steroids, mycophenolate, and biologic agents, may affect sleep through mood alterations, infection risk, muscle weakness, or metabolic side effects [64]. Pulmonary rehabilitation and structured exercise programs remain highly beneficial, improving sleep architecture, stabilizing autonomic balance, and enhancing oxygenation during exertion, all of which can indirectly contribute to better nocturnal breathing and improve tolerance to PAP therapy [49].

## 8 Conclusions

The coexistence of interstitial lung disease and obstructive sleep apnea represents a clinically significant and increasingly recognized intersection. ILD patients exhibit uniquely high rates of OSA driven not by classic risk factors such as obesity, but by restrictive mechanics, impaired ventilatory control, persistent inflammation, and severe nocturnal hypoxemia. Polysomnography in ILD reveals a distinctive pattern of disproportionate desaturation, REM-related vulnerability, sleep fragmentation, and altered architecture that is frequently missed by standard screening tools.

Accumulating evidence indicates that untreated OSA may adversely affect ILD outcomes—including accelerated lung function decline, increased risk of acute exacerbations, development or worsening of pulmonary hypertension, impaired quality of life, and higher mortality. Conversely, PAP therapy, when carefully implemented and supported, can improve sleep quality, daytime symptoms, and potentially clinical disease trajectories. Supplemental oxygen remains central in addressing pronounced nocturnal hypoxemia, while integrated management of comorbidities such as GERD, cough, and pulmonary hypertension optimizes results.

Despite these advances, significant gaps remain. Mechanistic human studies, large prospective cohorts, and intervention trials are needed to clarify causal pathways and refine therapeutic strategies. Digital health technologies and multidisciplinary care models offer promising directions for earlier detection and more comprehensive management.

Overall, recognizing and treating OSA in ILD is essential. A proactive and systematic approach to sleep evaluation should be incorporated into routine ILD care, with particular attention to nocturnal hypoxemia and REM-related physiology. As evidence continues to grow, addressing sleep-disordered breathing may become an important component of improving symptoms, nocturnal oxygenation, functional status, and quality of life, while its effect on survival and ILD progression requires confirmation in prospective trials.

## Declaration of generative AI and AI-assisted technologies in the writing process

During the preparation of this work the authors used ChatGPT 5 in order to improve language and readability. After using this tool/service, the authors reviewed and edited the content as needed and take full responsibility for the content of the publication.

## CRediT authorship contribution statement

**Nesrin Ocal:** Writing – original draft. **Baran Balcan:** Writing – original draft. **Yüksel Peker:** Writing – original draft.

## Declaration of competing interest

The authors declare the following financial interests/personal relationships which may be considered as potential competing interests: Yuksel Peker reports financial support was provided by ResMed Foundation outside the submitted work. Nesrin Ocal and Baran Balcan declare no competing financial interests or personal relationships that could have appeared to influence the work reported in this paper.

## References

[1] Senaratna C.V. (2017). Prevalence of obstructive sleep apnea in the general population: a systematic review. Sleep Med Rev.

[2] Garvey J.F. (2015). Epidemiological aspects of obstructive sleep apnea. J Thorac Dis.

[3] Cheng Y., Wang Y., Dai L. (2021). The prevalence of obstructive sleep apnea in interstitial lung disease: a systematic review and meta-analysis. Sleep Breath.

[4] Cardoso C.G. (2024). Obstructive sleep apnea in patients with fibrotic interstitial lung disease (non-idiopathic pulmonary fibrosis): what should be offered?. J Bras Pneumol.

[5] Khor Y.H. (2021). Interstitial lung disease and obstructive sleep apnea. Sleep Med Rev.

[6] Locke B.W., Lee J.J., Sundar K.M. (2022). OSA and chronic respiratory disease: mechanisms and epidemiology. Int J Environ Res Publ Health.

[7] Wei C.R. (2024). Exploring the prevalence and characteristics of obstructive sleep apnea among idiopathic pulmonary fibrosis patients: a systematic review and meta-analysis. Cureus.

[8] Saketkoo L.A. (2021). A comprehensive framework for navigating patient care in systemic sclerosis: a global response to the need for improving the practice of diagnostic and preventive strategies in SSc. Best Pract Res Clin Rheumatol.

[9] Karabul E. (2022). The frequency of obstructive sleep apnea in patients with primary Sjogren's syndrome. Sleep Breath.

[10] Martins R.B. (2023). Sleep parameters in patients with chronic hypersensitivity pneumonitis: a case-control study. J Bras Pneumol.

[11] Mariano P., Genta P.R. (2024). One step forward in understanding sleep in hypersensitivity pneumonitis patients. J Bras Pneumol.

[12] Joerns E.K., Sparks J.A. (2024). Interstitial pneumonia with autoimmune features: aiming to define, refine, and treat. Rev Colomb Reumatol.

[13] Yabuuchi Y. (2022). Impact of sleep-related hypoventilation in patients with pleuroparenchymal fibroelastosis. Respir Res.

[14] Lau H.X. (2025). Lymphoid interstitial pneumonia without known cause: diagnostic work up and differential considerations. Respirol Case Rep.

[15] Margaritopoulos G.A. (2024). Overnight desaturation in interstitial lung diseases: links to pulmonary vasculopathy and mortality. ERJ Open Res.

[16] George C.F., Kryger M.H. (1986). Sleep in restrictive lung disease. Sleep.

[17] Khor Y.H. (2021). Nocturnal hypoxaemia in interstitial lung disease: a systematic review. Thorax.

[18] Lancaster L.H. (2009). Obstructive sleep apnea is common in idiopathic pulmonary fibrosis. Chest.

[19] Troy L., Corte T. (2014). Sleep disordered breathing in interstitial lung disease: a review. World J Clin Cases.

[20] Bonsignore M.R. (2024). REM sleep obstructive sleep apnoea. Eur Respir Rev.

[21] Zhang X.L. (2019). Obstructive sleep apnea in patients with fibrotic interstitial lung disease and COPD. J Clin Sleep Med.

[22] Laz N.I. (2024). Study of the prevalence and predictive factors of sleep-disordered breathing in patients with interstitial lung diseases. The Egyptian Journal of Bronchology.

[23] Corte T.J. (2012). Elevated nocturnal desaturation index predicts mortality in interstitial lung disease. Sarcoidosis Vasc Diffuse Lung Dis.

[24] Yasuda Y. (2021). Analysis of nocturnal desaturation waveforms using algorithms in patients with idiopathic pulmonary fibrosis. Sleep Breath.

[25] Simonson J.L. (2022). Sleep architecture in patients with interstitial lung disease with and without pulmonary hypertension. Sleep Breath.

[26] Milioli G. (2016). Sleep and respiratory sleep disorders in idiopathic pulmonary fibrosis. Sleep Med Rev.

[27] Mermigkis C., Bouloukaki I., Schiza S. (2013). Obstructive sleep apnea in patients with interstitial lung diseases: past and future. Sleep & breathing = Schlaf & Atmung.

[28] Agarwal S. (2009). Interstitial lung disease and sleep: what is known?. Sleep Med.

[29] Valecchi D. (2023). Prognostic significance of obstructive sleep apnea in a population of subjects with interstitial lung diseases. Pulm Ther.

[30] Balcan B. (2024). Obstructive sleep apnea and pulmonary hypertension: a chicken-and-egg relationship. J Clin Med.

[31] Abboud F., Kumar R. (2014). Obstructive sleep apnea and insight into mechanisms of sympathetic overactivity. J Clin Investig.

[32] Wong A.W. (2020). A cluster-based analysis evaluating the impact of comorbidities in fibrotic interstitial lung disease. Respir Res.

[33] Dhont S. (2022). Pulmonary hypertension in interstitial lung disease: an area of unmet clinical need. ERJ Open Research.

[34] Ang H.L. (2024). Pulmonary hypertension in interstitial lung disease: a systematic review and meta-analysis. Chest.

[35] Shah F.A. (2021). Obstructive sleep apnea and pulmonary hypertension: a review of literature. Cureus.

[36] Louise B.J., Carys F., Philip M. (2020). Narrative review of sleep and pulmonary hypertension. J Thorac Dis.

[37] Sonti R. (2019). Multimodal noninvasive prediction of pulmonary hypertension in IPF. Clin Respir J.

[38] Myall K.J. (2023). Nocturnal hypoxemia associates with symptom progression and mortality in patients with progressive fibrotic interstitial lung disease. Chest.

[39] Sun X., Luo J., Xiao Y. (2014). Continuous positive airway pressure is associated with a decrease in pulmonary artery pressure in patients with obstructive sleep apnoea: a meta-analysis. Respirology.

[40] Arias M.A. (2006). Pulmonary hypertension in obstructive sleep apnoea: effects of continuous positive airway pressure: a randomized, controlled cross-over study. Eur Heart J.

[41] Bordas-Martinez J. (2024). Treating sleep-disordered breathing of idiopathic pulmonary fibrosis patients with CPAP and nocturnal oxygen treatment. A pilot study. Respir Res.

[42] Papadogiannis G. (2021). Patients with idiopathic pulmonary fibrosis with and without obstructive sleep apnea: differences in clinical characteristics, clinical outcomes, and the effect of PAP treatment. J Clin Sleep Med.

[43] Adegunsoye A. (2020). CPAP adherence, mortality, and progression-free survival in interstitial lung disease and OSA. Chest.

[44] Mermigkis C., Bouloukaki I., Schiza S.E. (2017). Sleep as a new target for improving outcomes in idiopathic pulmonary fibrosis. Chest.

[45] Patil S.P. (2019). Treatment of adult obstructive sleep apnea with positive airway pressure: an American academy of sleep medicine systematic review, Meta-Analysis, and GRADE assessment. J Clin Sleep Med.

[46] Mermigkis C. (2012). CPAP treatment in patients with idiopathic pulmonary fibrosis and obstructive sleep apnea--therapeutic difficulties and dilemmas. Sleep Breath.

[47] Bordas-Martinez J. (2024). Treating sleep-disordered breathing of idiopathic pulmonary fibrosis patients with CPAP and nocturnal oxygen treatment. A pilot study : Sleep-disordered breathing treatment in IPF. Respir Res.

[48] Clark K.P. (2023). Supplemental oxygen therapy in interstitial lung disease: a narrative review. Ann Am Thorac Soc.

[49] Dowman L., Hill C.J., Holland A.E. (2014). Pulmonary rehabilitation for interstitial lung disease. Cochrane Database Syst Rev.

[50] Sunwoo B.Y., Malhotra A. (2024). Mechanical interactions between the upper airway and the lungs that affect the propensity to obstructive sleep apnea in health and chronic lung disease. Sleep Med Clin.

[51] Koo P. (2017). Change in end-expiratory lung volume during sleep in patients at risk for obstructive sleep apnea. J Clin Sleep Med.

[52] Eckert D.J., Malhotra A. (2008). Pathophysiology of adult obstructive sleep apnea. Proc Am Thorac Soc.

[53] Aboussouan L.S., Mireles-Cabodevila E. (2017). Sleep-disordered breathing in neuromuscular disease: diagnostic and therapeutic challenges. Chest.

[54] Boentert M. (2023). Sleep disorders in neuromuscular diseases: a narrative review. Clinical and Translational Neuroscience.

[55] Antonaglia C. (2024). Deciphering loop gain complexity: a primer for understanding a pathophysiological trait of obstructive sleep apnea patients. Respir Med.

[56] Ryan S., Taylor C.T., McNicholas W.T. (2005). Selective activation of inflammatory pathways by intermittent hypoxia in obstructive sleep apnea syndrome. Circulation.

[57] Dogan D. (2016). Assessment of the role of serum ischemia-modified albumin in obstructive sleep apnea in comparison with interleukin-6. Postgrad Med J.

[58] Lavie L. (2003). Obstructive sleep apnoea syndrome--an oxidative stress disorder. Sleep Med Rev.

[59] Jelic S. (2008). Inflammation, oxidative stress, and repair capacity of the vascular endothelium in obstructive sleep apnea. Circulation.

[60] Mochol J. (2021). Cardiovascular disorders triggered by obstructive sleep Apnea—A focus on endothelium and blood components. Int J Mol Sci.

[61] Kapur V.K. (2017). Clinical practice guideline for diagnostic testing for adult obstructive sleep apnea: an American academy of sleep medicine clinical practice guideline. J Clin Sleep Med.

[62] Rochwerg B. (2017). Official ERS/ATS clinical practice guidelines: noninvasive ventilation for acute respiratory failure. Eur Respir J.

[63] Richeldi L. (2014). Efficacy and safety of nintedanib in idiopathic pulmonary fibrosis. N Engl J Med.

[64] Fischer A., du Bois R. (2012). Interstitial lung disease in connective tissue disorders. Lancet.

[65] Bouloukaki I. (2022). Overlaps between obstructive sleep apnoea and other respiratory diseases, including COPD, asthma and interstitial lung disease. Breathe.

[66] Schiza, S., et al., Idiopathic pulmonary fibrosis and sleep disorders: no longer strangers in the night. Eur Respir Rev. 24(136): p. 327-339. 10.1183/16000617.00009114 PMC9487812 26028644

[67] Faverio P. (2019). Management of chronic respiratory failure in interstitial lung diseases: overview and clinical insights. Int J Med Sci.

